# Modulation of Apoptosis by Cytotoxic Mediators and Cell-Survival Molecules in Sjögren’s Syndrome

**DOI:** 10.3390/ijms19082369

**Published:** 2018-08-11

**Authors:** Hideki Nakamura, Yoshiro Horai, Toshimasa Shimizu, Atsushi Kawakami

**Affiliations:** 1 Department of Immunology and Rheumatology, Division of Advanced Preventive Medical Sciences, Nagasaki University Graduate School of Biomedical Sciences, 1-7-1 Sakamoto, Nagasaki City, Nagasaki 852-8501, Japan; toshimasashimizu2000@yahoo.co.jp (T.S.); atsushik@nagasaki-u.ac.jp (A.K.); 2 Clinical Research Center, National Hospital Organization Nagasaki Medical Center, Kubara 2-1001-1, Omura 856-8562, Japan; horaiy@nagasaki-mc.com

**Keywords:** Sjögren’s syndrome, apoptosis, Fas, TLR, EGF, salivary gland epithelial cells, cell survival molecule

## Abstract

The pathogenesis of Sjögren’s syndrome (SS) involves multiple factors including genetic background, cell death, and exocrine dysfunction. We here discuss apoptotic control in exocrine glands in SS by showing various pro- and anti-apoptotic pathways. Although the membrane-bound and soluble form of the Fas/Fas ligand system is a leading player with activation of the death domain and caspase 8/3 cleavage, the role of soluble Fas/FasL (including its polymorphism) in apoptosis is controversial. The tumor necrosis factor related apoptosis-inducing ligand (TRAIL)-mediated apoptosis of salivary gland epithelial cells (SGECs) involves a mitochondrial pathway that includes caspase 9 cleavage. The involvement of innate immunity cells such as toll-like receptors (TLRs) has been investigated; TLR2-4 and TLR7-9 are associated with the induction of inflammation in exocrine glands of SS patients. TLR3 has the potential to induce the apoptosis of SS patients’ SGECs. Linkage of epidermal growth factor (EGF) was shown in exocrine glands in SS, and it inhibited the Fas/FasL system with the help of cell-survival factors. TLR3 has dual actions to cause inflammation as well as apoptosis, which are inhibited by EGF. In conclusion, apoptosis in exocrine glands of SS patients is tightly controlled by balance of pro-apoptotic signals and growth factor.

## 1. Introduction

Sjögren’s syndrome (SS) is an autoimmune disease characterized by sicca symptoms including xerophthalmia and xerostomia, extraglandular manifestations such as interstitial pneumonia and interstitial nephritis, and the appearance of autoantibodies such as anti-Ro/SS-A, La/SS-B antibodies [1,2,3,4,5]. Because of the activation of the B-cell system in concert with T cells that respond to autoantigens, T or B cell-targeting therapies including abatacept, rituximab, and belimumab are possible beneficial biologics for the treatment for SS [6,7,8,9]. With regard to SS patients’ genetic backgrounds, genome-wide association studies (GWAS) for SS patients showed susceptible loci in major histocompatibility complex and in regions for GTF21, along with differences among ethnic groups [10,11]. Although interferon-γ and Th2 cytokines are pathologically major factors for T cell-related immunological dysfunction in SS [12,13], a recent study showed the importance of the involvement of Th17 cells in SS [14].

In contrast, other studies showed that B-cell activation in the ectopic germinal center (GC) was also mediated by C-X-C motif chemokine 13 (CXCL13), and that B-cell activating factor of the tumor necrosis factor (TNF) family (BAFF) is also involved in the pathology of SS [15,16,17]. CD40-CD40 ligand expression on lymphocytes is also involved in T-cell activation after T–B cell interaction [18,19]. Although these issues are recent topics in SS research, the cell death of salivary and lacrimal gland epithelial cells and its control have been long-standing topics for discussion [20,21]. Several molecules are reported to be connected to the apoptosis of epithelial cells in SS. However, the whole picture of regulatory factors that act to inhibit the apoptosis of salivary gland epithelial cells (SGECs) remains to be established.

Another crucial point in the pathogenesis of SS is the epithelial cell activation by innate immunity cells such as toll-like receptors (TLRs) [22,23] since the type I interferon (IFN) signature including the activation of IFN-α or IFN-β might be involved in the pathogenesis of SS. Because type I IFN (which is activated in plasmatoid dendritic cells [pDCs] in peripheral blood) is suspected to be triggered by viral infection [24,25], inflammation triggered by innate immunity is one of unsolved issues in SS. It was demonstrated that TLR-mediated apoptosis [26,27] is also involved in SS as a novel pattern of cell death. In this review article, we discuss the apoptotic process mediated by the Fas system, TRAIL, and TLRs, and we consider the regulation of this process (which is performed by soluble factors such as growth factor) in reference to the involvement of TLRs in the pathogenesis of SS.

## 2. Fas and Protease-Mediated Apoptosis in Sjögren’s Syndrome

### 2.1. Membrane-Bound Fas and Apoptosis in Individuals with SS

Fas (also called APO-1, APT, CD95) [28,29,30], which is known as an apoptosis receptor and a member of the TNF receptor superfamily, induces programmed cell death, i.e.; apoptosis. In contrast, Fas ligand (FasL) [31,32,33], which belongs to the TNF family as a type II transmembrane protein, binds with Fas at death-inducing signaling complex (DISC) and induces apoptosis following the assembly of Fas-associated protein with death domain (FADD) and the subsequent activation of cysteine-proteases.

**Figure 1:**
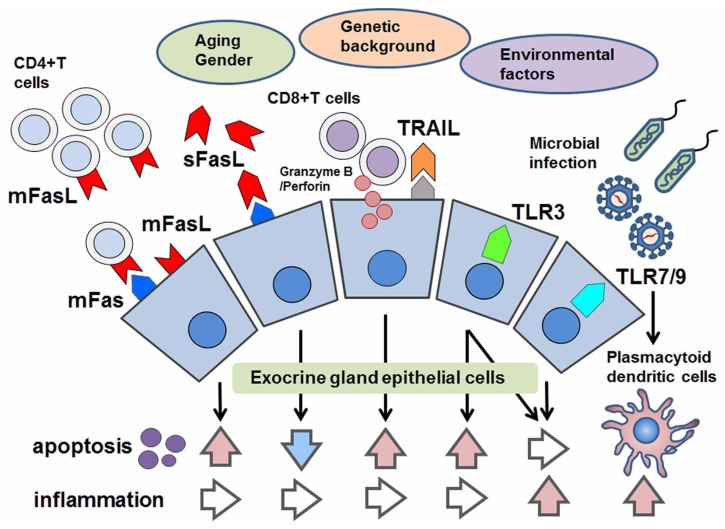
Schematic overview of apoptotic control in Sjögren’s syndrome (SS). With respect to apoptosis in SS, aging, gender, genetic factors and environmental factors have been considered. As pro-apoptotic factors, the following are effectors to induce apoptosis in epithelial cells: the joining of membranous Fas (mFas) that expresses on epithelial cells and Fas ligand (mFasL) expressed on infiltrating CD4^+^T lymphocytes; cytotoxic granules such as granzyme B/Perforin in CD8^+^T cells that are adjacent to epithelial cells; TRAIL and the stimulation of toll-like receptor3 (TLR3). Even though TLR3 has the potential to induce apoptosis, TLR3 and TLR7–9 mediate inflammation in response to microbial infection. In contrast, the soluble form of FasL (sFasL) is presumed to inhibit mFas-mediated apoptosis, in which some modifications with respect to the promotor polymorphism of the soluble form of Fas/FasL might be associated with their function in apoptotic regulation.

Fas/FasL system-mediated apoptosis is now known to have a crucial position in the immune system as well as cancer immunity [34]. Ichikawa et al. [35] reported that Fas expression was detected in CD4^+^T cells among peripheral blood mononuclear cells (PBMCs) of patients with primary SS. In contrast to this Fas expression, the expression of bcl-2 protein was reduced, suggesting that an imbalance of Fas/bcl-2 is one of the characteristics of PBMCs in primary SS. Kong et al. reported Fas and FasL expression in salivary glands of patients with SS (Figure 1) [36]. Although Fas^+^FasL^+^ acinar cells showed apoptosis as DNA fragmentation, Kong et al. also reported that bcl-2 showed a diminished intracellular expression pattern in CD4^+^T cells. Although an investigation by our group [37] also revealed the expression of Fas and FasL in acinar cells of labial salivary glands (LSGs), apoptosis detected by DNA fragmentation by using nick end labelling (TUNEL) was not frequent compared to the expression of Fas antigen. In addition, Fas expression was observed on the luminal side of ducts, suggesting that a Fas–FasL interaction might occur on the lumen side of ducts.

Regarding FasL expression in infiltrating cells, Matsumura et al. [38] also confirmed of Fas/FasL system in interstitial nephritis of SS patients which showed Fas-mediated apoptosis was involved in organ damage in SS. With regard to the mechanism underlying Fas-mediated apoptosis in SS, we showed that anti-Fas antibody induced apoptosis in SGECs obtained from patients with SS in connection with the cleavage of caspase 8 and caspase 3 without the afore-mentioned cleavage of caspase 9 [39]. Interestingly, the Fas-mediated apoptosis in SGECs required LY294002 (a selective phosphoinositide 3-kinase (PI3K) inhibitor), indicating that SGECs might be protected from various apoptosis-inducing factors. Because the effect of LY294002 is known to be mediated through the inhibition of PI3K/Akt/mammalian target of Rapamycin (mTOR) [40], these cell-survival factors are thought to produce an effective action as inhibitors of apoptosis in SS.

A similar effect of Fas toward apoptosis in SS was also described in association with CD40 signaling [41]. It was reported that stimulation with anti-Fas antibody for IFN-γ-pre-incubated SGECs obtained from SS patients had no apoptotic effect without the cooperation of anti-CD40 antibody. Those authors concluded that CD40 had the potential to promote Fas-mediated apoptosis toward SGECs by a down-regulation of an inhibitory molecule, c-FLIP. These findings suggested that stimulation with Fas alone might not have enough effect to induce apoptosis in primary cultured SGECs.

With regard to salivary gland-derived cells, the sensitivity to anti-Fas antibody differed between primary cultured SGECs and several cell lines. HSY cells [42] are derived from a human parotid gland adenocarcinoma cell line. When HSY cells were treated with anti-Fas antibody, time-dependent increases in the cells’ levels of μ-calpain and caspase 3 were observed [43]. In addition, an up-regulation of 120-kD α-fodrin [44,45,46,47] (which is known as an SS-related autoantigen) was detected in HSY cells that were transfected with full-length caspase 3 and μ-calpain cDNA, suggesting that α-fodrin plays a crucial role in Fas-mediated apoptosis in SS. Regarding FasL in lacrimal glands from individuals with SS, it was reported that a down-regulation of FasL was closely associated with an enlargement of lacrimal glands in SS [48], and that the down-regulation of FasL in SS patients with enlarged lacrimal glands was induced by a point mutation of FasL gene promoter.

### 2.2. Membrane-Bound Fas and Apoptosis in a Murine Model of SS

Several studies have examined Fas-mediated apoptosis in a murine model of SS. Salivary glands of interleukin (IL)-10 transgenic (Tg) mice were reported to exhibit TUNEL-positive apoptosis on epithelial ducts and acinar cells in association with Fas expression [49]. Interestingly, FasL was expressed in CD4^+^T cells but not epithelial cells in IL-10 Tg mice. In addition, in vitro spleen CD4^+^T cells treated with recombinant IL-10 dose-dependently expressed FasL. Moreover, recombinant IL-10 with concanavalin A and recombinant IL-2 enhanced cytotoxic activity toward SGECs, suggesting that IL-10 has the potential to up-regulate the Fas/FasL system for apoptotic induction.

With regard to the regulatory mechanisms of Fas-mediated apoptosis in a murine model, Ishimaru et al. [50] demonstrated that estrogen deficiency could be a candidate to enhance Fas-mediated glandular destruction in their murine SS model. They showed SS-like severe sialadenitis with TUNEL-positive apoptosis in estrogen-deficient mice, and they observed that estrogen administration blocked this autoimmune lesion. Interestingly, the Fas-mediated apoptosis of cultured SGECs was inhibited by estrogen administration in vitro. Ishimaru et al. subsequently reported [51,52] that aged NFS/*sld* mice showed decreased saliva secretion, tear flow, and TUNEL-positive severe destructive lesions in their salivary glands compared to those in younger mice. In the aged mice, the frequency of the SGECs with Fas was much higher than that in younger mice, although the frequency of FasL-positive CD4^+^T cells in salivary glands was similar between the two groups. On the other hand, paradoxical Fas expression in spleen CD4^+^T cells was found in the aged mice, suggesting that the Fas-mediated apoptosis in aged mice was closely related to the dysregulation of CD4^+^T cells.

With regard to the lacrimal involvement in an SS murine model that showed FasL expression in lacrimal glands, an effect of an immunosuppressive agent, cyclosporine A (CyA) was demonstrated [53]. In association with decreases of apoptosis and FasL in infiltrating lymphocytes, the administration of CyA improved tear function in NFS/sld mice. In NOD mice, FasL in infiltrating lymphocytes was also reduced with an improvement of tear function as an effect of CyA, suggesting that a direct effect of CyA toward FasL occurred in lacrimal lesions.

Regarding the involvement of lymphocytes in salivary glands, an interesting investigation was conducted using NOD-scid mice [54]. Specifically, it was demonstrated that NOD-scid mice with SS-like pathology without infiltrations of T and B lymphocytes showed TUNEL-positive apoptosis in their salivary epithelial cells, suggesting that apoptosis might be induced by the Fas/FasL system without the engagement of lymphocytes. Another study examined the pharmacological effect of total glucosides of peony (TGP), which has been empirically used as a treatment for SS, and the effects of TGP on the expressions of Fas and FasL in NOD mice that showed SS-like symptoms were revealed [55]. Without TGP treatment, control NOD mice showed increased expressions of IFN-γ, IL-4, Fas and FasL, whereas the expressions of these molecules were reduced in TGP-treated mice and in mice treated with both TGP with hydroxychloroquine. Those results suggested that the administration of TGP has the potential to improve the Th1/Th2 cytokine balance and the Fas/FasL system.

## 3. Soluble Fas/FasL and Apoptosis in Sjögren’s Syndrome

In addition to membranous Fas, another isoform is known: soluble Fas (sFas) [56]. Similar to sFas, membranous-type FasL is also changed to a soluble form (sFasL) [57] that is cleaved at a conserved cleavage site by matrix metalloproteinase 7. A commonly accepted theory is that a function of sFas is protective action toward apoptosis, whereas sFasL may be a pathological molecule that induces tissue damage [57]. In terms of autoimmune diseases, the functions and clinical significance of sFas and sFasL have not been clearly determined. Nozawa et al. [58] reported that the level of sFas in the sera of patients with autoimmune diseases (including systemic lupus erythematosus (SLE), polymyositis/dermatomyositis (PM/DM), mixed connective tissue disease (MTCD), and SS) was significantly higher than that in healthy subjects. They also reported that sFasL in sera was especially high in patients with SS, suggesting that the sFas/sFasL system might be associated with the glandular destruction in SS. Our study [59] revealed high levels of sFasL in saliva obtained from SS patients in a glandular-destructive group, based on the sialography described by Rubin and Holt [60]. Although these studies suggested that sFas/FasL might play two different roles, i.e., destructive and protective roles toward apoptosis.

However, a recent study [61] showed that the serum levels of sFasL in patients with SS were significantly lower than those of healthy subjects, and the sFasL levels of the patients in the severe disease activity group were lower than those of the patients in the mild disease activity group. Interestingly, that investigation also revealed that the serum IL-10 level was positively related to the serum sFasL level. Since IL-10 has a rather anti-inflammatory or protective action toward glandular injury in SS, it is possible that sFasL works with IL-10 to inhibit tissue damage. A study using a murine model of SS indicated that soluble anti-FasL antibody, FLIM58 induced destructive autoimmune lesions with an elevation of serum antibodies against 120-kD α-fodrin [62], suggesting that (at least in that murine model of SS) the generation of sFasL had the potential to inhibit activation-induced cell death and the subsequent increase of CD4^+^T cells.

With respect to the relationship between the polymorphism of Fas and sFas in primary Sjögren’s syndrome (pSS), FAS-670A > G promotor polymorphism was studied [63] in association with the serum level of sFas. This polymorphism is also suspected to delete autoreactive lymphocytes. Interestingly, the patients in that study bearing the AA genotype showed high levels of sFas compared to those with the GG genotype, although there was no association between this promotor polymorphism and the serum sFas level. In addition, the pSS patients with A allele were closely associated with an elevated level of sFas in pSS, suggesting that not only the sFas level but also genetic alterations might modify the process of the sFas-mediated inhibition of apoptosis.

### Cytotoxic Granules and Apoptosis in SS

Granzyme B is a type of serine protease that was originally detected in the granules of cytotoxic T cells or natural killer cells [64]. With respect to the apoptosis of alloreactive cytotoxic T cells, it was reported that granzyme B was important in an experiment using granzyme B-deficient mice [65]. Regarding the action of proteases, granzyme B-mediated cleavage of autoantigen has been shown to produce autoantibodies that recognized La/SS-B protein [66]. IL-21 was shown to have the potential to induce B cells that had granzyme B activity [67], and it was reported that T follicular helper (Tfh)-mediated IL21 expression induced granzyme B in CD5^+^B cells in SS [68], suggesting that as a cytotoxic serine protease, granzyme B is regulated by Tfh-mediated IL-21 production.

In an investigation of the expression of perforin, which provides the cytotoxicity of cytotoxic T cells [69,70], Fas/FasL and granzyme B/perforin were examined in lacrimal glands obtained from SS patients and healthy subjects [71]. Fas was expressed on the acinar cells in lacrimal glands obtained from both the SS patients and healthy subjects, although FasL was expressed only on the acinar cells in lacrimal glands from the SS patients. Intriguingly, the epithelial cells surrounded by CD8^+^T cells with alpha E beta 7 were apoptotic, suggesting that a unique expression of CD8^+^T cells that had cytotoxic granules such as granzyme B/perforin also induces apoptosis.

## 4. TRAIL-Mediated Apoptosis in Sjögren’s Syndrome

TNF-related apoptosis-inducing ligand (TRAIL; CD253, TNFSF10) [72,73,74], a member of the TNF ligand family, is also known to induce the apoptosis of target cells. One of the characteristics of TRAIL is its binding to death receptor (DR) 4/5, after which caspase 8-dependent apoptosis is induced. After TRAIL binds to DR4/5, an extrinsic pathway that includes Fas-associated protein with death domain (FADD) and caspase8/3 cleavage induces apoptosis.

As a unique apoptotic system of TRAIL, the release of cytochrome C after the cleavage of Bit is followed by the cleavage of caspase 9, resulting in caspase 3 cleavage as an intrinsic pathway. With respect to the expression of TRAIL in LSGs of patients with SS, it was reported that TRAIL was expressed on mononuclear cells (MNCs) at massive infiltrating sites, whereas TRAIL receptors (including TRAIL-R1/2) were expressed on ductal epithelial cells [75]. By using a human salivary duct cell line (HSG), those researchers also showed that IFN-γ induced TRAIL-R1 on HSG cells that were sensitive to recombinant TRAIL protein, resulting in a lapse into apoptosis. Chen et al. observed a high expression of TRAIL in infiltrating MNCs of grade IV SS patients with anti-SS-A antibody positivity and a higher titer of antinuclear antibody [76]. In addition, overexpressions of TRAIL, metalloproteinase-3, and intercellular adhesion molecule-1 were associated with a more severe grade of LSGs biopsy, suggesting that TRAIL might be associated with the degree of inflammation in LSGs.

With respect to viral infection, there is an interesting report regarding the relationship between TRAIL and natural killer (NK) cells in which the NK cells eliminated activated CD4^+^T cells in cytomegalovirus infection [77]. Although this elimination was dependent on NK cell-mediated TRAIL expression, the deletion of CD4^+^T cells induced a chronic infection state that was similar to the focal lymphocytic infiltration observed in the LSGs of patients with SS. Regarding the mechanism of TRAIL-mediated apoptosis of SGECs obtained from individuals with SS, we demonstrated that compared to Fas-mediated apoptosis, TRAIL-mediated apoptosis occurred faster during a 3-h stimulation by recombinant TRAIL [39]. In addition, the apoptosis was accompanied by caspase 9 cleavage, suggesting that TRAIL-mediated apoptosis occurred through a mitochondrial pathway. The involvement of this pathway was based on the co-expression of cytochrome release and the activation of Apaf1. However, careful consideration is necessary with regard to the TRAIL-mediated apoptosis of SGECs, because this apoptosis involving the mitochondrial pathway was also observed in SGECs obtained from non-SS subjects.

## 5. The Involvement of TLRs in Sjögren’s Syndrome

### 5.1. TLRs and Autoimmune Diseases

Innate immunity has been shown to be involved in the recognition of pathogens such as bacteria, virus and parasites. Although the retinoic acid-inducible gene I (RIG-I) receptors (RLRs) [78] and NOD-like receptors (NLRs) [79] have crucial roles in innate immunity, toll-like receptors (TLRs) [80,81,82] are the most important molecules for the recognition of pathogenic microbes. TLRs have a type I transmembrane structure [83] and play a crucial role in the determination of the response toward microbes with regard to immune cells including dendritic cells, macrophages and B cells. The leucine-rich repeat (LRR) in the extracellular domain of TLRs is an essential factor for recognizing ligands.

Fourteen species of TLRs in mammals, including at least 10 species of TLRs in human are known, and some of the known TLRs are associated with autoimmune diseases. Abdollahi-Roodsaz et al. reported that IL-1 receptor antagonist (*IL-1rn^−/−^*) knockout mice showed severe arthritis with reduced Tregs function and an up-regulation of IFN-γ production, whereas *IL-1rn^−/−^Tlr4^−/−^* mice showed a suppression of severe arthritis with reduced numbers of Th17 cells [84]. In addition, the knockout of TLR9 resulted in no progression of arthritis, suggesting that these three TLRs (i.e., TLR2, TLR4, and TLR9) have different relationships with the induction of arthritis and IL-17 production. Abdollahi-Roodsaz et al. also reported that TLR2 deficiency augmented the severity of rheumatoid arthritis (RA)-like arthritis with inflammatory joint and bone erosion by modulating the Fcγ receptor of macrophages, indicating a clear inhibitory effect of TLR2 toward arthritis [85].

Regarding the relationship between RA and TLR7, a strong correlation between the disease activity of RA and the TNF-α level was demonstrated [86]. In contrast, TLR3 expression was observed in glomerular mesangial cells in lupus model mice, i.e., MRL*lpr/lpr* mice [87], although TLR3, 7 and 9 expressed in infiltrating macrophages in these mice with nephritis. Different roles of TLR7 and TLR9 were indicated by Christensen et al., who reported that TLR9^−/−^ mice showed an exacerbation of lupus activities including the activation of lymphocytes, i.e., plasmatoid dendritic cells (pDCs) with an upregulation of IFN-α activity, in contrast to the pathologic condition observed in TLR7^−/−^ mice [88]. The same research group subsequently demonstrated that the manifestations in TLR9^−/−^ mice that showed exacerbated lupus symptoms depended on TLR7, and that TLR9 inhibited the autoantibody production that was dependent on TLR7 [89]. In contrast, TLR7 was needed for the organomegaly that was observed in TLR9^−/−^ mice, indicating that TLR7 and TLR9 worked together to control clinical symptoms and autoantibody production in lupus.

With regard to the relationship between the aforementioned FasL and TLRs, a recent study showed that FasL had the effect of exacerbatting TLR7-dominant lupus model mice [90]. In multiple sclerosis, the role of IFN-β in the activation of TLR7 in pDCs was demonstrated [91]. In PBMCs obtained from patients with multiple sclerosis, IFN-β upregulated the mRNA of MyD88, TLR3, and TLR7, whereas TLR9 mRNA expression was suppressed. Although pDCs expressed TLR7/9 in the endosome, the administration of IFN-β upregulated the expression of TLR7 in pDCs. In addition, loxoribine (an agonist of TLR7) increased the IFN-α production in PBMCs pretreated with IFN-β, suggesting that the activation of TLR7 induces the production of type I IFN in multiple sclerosis.

### 5.2. TLRs in Sjögren’s Syndrome

#### 5.2.1. TLR1–4 in SS

With respect to TLR1–4, results from quantitative real-time polymerase chain reaction (RT-PCR) analyses using cultured SGECs obtained from patients with and without SS showed dominant expressions of TLR1, 2, and 4 in SS [92]. In addition, TLR ligands including peptidoglycan (TLR2), polyinosinic:cytidylic acid (polyI:C) (TLR3), and lipopolysaccharide (LPS) (TLR4) induced various levels of intercellular adhesion molecule (ICAM)-1, CD40, B7-2, and major histocompatibility complex (MHC)-I in SGECs from SS patients, suggesting that epithelial cells of SS have a functional action in response to innate immunity. We also demonstrated that TLR2-4 and myeloid differentiation factor (MyD)88 were expressed in LSGs of SS patients compared to those of non-SS subjects [93], in which non-SS subjects showed weak expression of these molecules. We also observed that the stimulation of a human salivary gland (HSG) cell line with polyI:C or LPS induced the expression of CD54 along with the phosphorylation of extracellular signal-regulated kinase (ERK), c-jun-N-terminal kinase (JNK), and p38.

The relationship between virus infection and TLR3 stimulation in SS has also been examined. PolyI:C, a synthetic agonist for TLR3 with reovirus-1 (which is a type of double-strand RNA virus) induced a strong expression of BAFF mRNA and the secretion of BAFF protein from SGECs [94]. This BAFF expression was partially inhibited by anti-IFN-α receptor 1 antibody, suggesting that there was an IFN-α-independent pathway in BAFF expression after stimulation with TLR3 ligand and virus. Investigations of the involvement of TLR in the activation of Th17 cells (which have the potential to produce IL-17) identified TLR2 ligation and LSGs’ expressions of TLR2, TLR4, TLR6 and IL-17 [95,96]. TLR2, TLR4 and TLR6 were expressed on both infiltrating mononuclear cells and epithelial cells. In addition, IL-17 expressed on CD4^+^T cells was dominantly observed in both PBMCs and LSGs from patients with pSS compared to healthy subjects. It was also shown that TLR2 stimulation of PBMCs of patients with pSS especially produced IL-17 and IL-23, with the involvement of signal transducer and activator of transcription 3 (STAT3) and nuclear factor-kappa B (NF-κB) pathways [95,96].

#### 5.2.2. TLR7–9 in Sjögren’s Syndrome and SLE

Among the TLRs, the aforementioned TLR1, 2 and 4 exist on the cell surface, whereas TLR3 and TLR7–9 (which recognize nucleic acids) exist in the endoplasmic reticulum or endosome [97,98]. With regard to species of nucleic acids, TLR7 and TLR8 respond to single-strand RNA [99,100], and TLR3 responds to double-strand RNA [101,102]. When TLR7/9 in pDCs are activated by recognition of viruses, two pathways are activated: a type I IFN pathway with the production of IFN-α and IFN-β [103] and the NF-κB pathway, which induces TNF-α or IL-6 [104]. The expressions of TLR7–9 were described by Zheng et al. to be primarily in PBMCs and parotid glands of patients with pSS [105], and in PBMCs, the TLR7 and TLR9 mRNA levels were significantly higher than those of healthy subjects despite similar levels of TLR8 in the pSS and healthy groups. Regarding the expressions of TLR7–9 in parotid glands, TLR7 and TLR9 were expressed on the epithelial islands and ductal epithelial cells as well as infiltrating lymphocytes. Zheng et al. also noted that TLR7 and TLR9 were expressed in ductal epithelial cells of healthy subjects. As for Ro/SS-A in SS and TLR7/9, a relationship between Ro52 and IRF7 (which is a crucial downstream factor for TLR7/9-mediated IFN-α production) was reported [106]. Specifically, it was shown that Ro52 ubiquitinated and inhibited IRF7 by a negative regulation for IRF7 as a type of degradation, indicating that the degradation of IRF7 might lead to TLR7 and TLR9 stimulation.

Karlsen et al. reported TLR7 and TLR9 in PBMCs obtained from patients with pSS [107]. Although they observed similar frequencies of CD19^+^B cells in pSS patients and control subjects, the numbers of naïve B cells and pre-switched memory B cells were increased in PBMCs from the patients with pSS. However, there was no significant difference with respect to the expression of TLR7 or TLR9 in B cells between pSS patients and control subjects. Karlsen et al. subsequently reported [108] the response of B cells to stimulation with TLR7 and TLR9; a low frequency of IL-10-positive pre-switched memory B cells was observed regardless of the presence of stimulation with TLR7 and TLR9, suggesting that the B-cell population and responsiveness in pSS patients differ from those in healthy subjects.

A recent examination of the relationship between type I IFN and pDCs in SS conducted a genome-wide microarray analysis of isolated pDCs and CD14^+^ monocytes obtained from 115 patients with pSS and 42 healthy subjects [109]. In accordance with the above-cited studies, TLR7 gene (but not TLR9 gene) was up-regulated when the cell types were restricted to IFN-positive cells. Interestingly, the expressions of RIG-I and IFIH1/melanoma differentiation associated gene-5 (MDA5) as well as those of MyD88 and IRF7 (which are downstream molecules of TLR7) were also enhanced. In addition, up-regulations of TLR7, RIG-I and IFIH/MDA5 were specifically detected in IFN-positive patients with pSS, suggesting that pDCs that express a TLR7 signaling pathway might be a candidate for treating pSS.

Regarding the role of type I IFN in autoimmune diseases including SLE and pSS, an involvement of endogenous virus-like genomic repeat elements was reported in a 2016 study [110] in which the expressions of type I IFN and the above-mentioned virus-related elements were examined by using LSGs obtained from patients with pSS and renal tissue obtained from patients with lupus nephritis. In both types of specimens, transcripts of long interspersed nuclear element 1 (LINE-1) were increased, and a correlation between LINE-1 and type I IFN was observed. In addition, the transfection of LINE-1-encoding nucleic acids toward pDCs or monocytes induced type I IFN. The production of LINE-1-mediated IFN-α in pDCs was diminished in accord with the administration of an inhibitor of TLR7/8, demonstrating that TLR7/8-associated viral elements had the potential to activate a type I IFN pathway in pSS and SLE. Related to TLR signaling, the necessity of MyD88 was recently reported by Kiripolsky et al., using a murine model of pSS [111]. They showed that NOD.B10Sn-*H2^b^* /J mice that lack *MyD88* had diminished inflammation and improved saliva flow. These *MyD88*-lacking mice also had splenocytes that showed no secretion of IL-6 after the administration of a MyD88-dependent agonist, indicating that MyD88 played a crucial role in the LR-mediated emergence of manifestations as well as cytokine production in SS.

We are also investigating the expressions of TLR7–9 in LSGs and the function of SGECs obtained from patients with pSS. We observed TLR7-dominant expression in infiltrating mononuclear cells (MNCs) and ductal epithelial cells and downstream molecules in their LSGs. In contrast, cultured SGECs stimulated with IFN-β and a TLR7 agonist produced no IFN-α compared to peripheral blood pDCs that produced IFN-α in response to TLR7 stimulation (unpublished data).

## 6. TLR3-Mediated Apoptosis in Sjögren’s Syndrome

TLR3 signaling usually promotes the activation of NF-κB pathway and IRF3 with pro-inflammatory cytokines [98]. In HepG2 (liver carcinoma) cells and hepatocellular carcinoma cells, TLR3 induced apoptosis without an induction of pro-inflammatory cytokines [112,113,114,115]. With regard to TLR3-mediated apoptosis in SS, Manoussakis et al. were the first to report a unique type of cell death in which morphologically TLR3 ligand poly I:C induced detachment-induced cell death, or so-called “anoikis” [116]. Manoussakis et al. also observed increased expressions of Bmf (a type of pro-apoptotic signal), BimEL, and Bax in contrast to a decreased expression of survival factor Bcl-2. Our study also demonstrated TLR3-mediated cell death in SGECs obtained from patients with pSS and healthy subjects, in which the TLR3 ligand poly I:C induced nuclear fragmentation that was revealed as apoptosis by TUNEL staining, whereas peptidoglycan and LPS induced no apoptosis of SGECs [117]. Interestingly, the TLR3-mediated apoptosis was augmented by our addition of a phosphoinositide 3-kinase, i.e., the PI3K inhibitor LY294002. In addition, the administration of poly I:C activated protein kinase/Jun-terminal kinase, p44/42 MAP kinase, and Akt. The research into TLR3-mediated apoptosis is limited, and further studies to elucidate the TLR3-mediated cell death machinery are required.

## 7. Control of Apoptosis in Sjögren’s Syndrome

There are several reports of in vivo and in vitro studies of the inhibition of Fas-mediated apoptosis in SS. Polihronis et al. [118] demonstrated the expressions of apoptosis-related molecules, including the expression of Bcl-2 on both ductal epithelial cells and infiltrating MNCs; the pro-apoptotic molecules Fas, FasL and Bax were also expressed. However, pS2 (a promotor of epithelial cell repair) was expressed on epithelial cells from patients with pSS, suggesting that an epithelial cell-specific expression of pS2 might be associated with the inhibition of epithelial cell apoptosis in pSS.

Concerning the frequency of apoptosis in SS, Ohlsson et al. [119] reported that Fas-mediated apoptosis revealed by TUNEL staining was rare regardless of the expression of FasL in infiltrating MNCs, suggesting the existence of an inhibitory mechanism for Fas-mediated apoptosis in SS. Regarding the inhibition of apoptosis in infiltrating MNCs, we observed the expressions of CD40 and CD40 ligand (CD40L) on MNCs in LSGs obtained from patients with SS [120]. The expression of CD40 on the MNCs co-existed with expressions of Bcl-2 or Bcl-x, which were also abundantly expressed on infiltrating MNCs, with a lower expression of Bax compared to that of BCl-2/Bcl-x. In another study, the expression of CD40 on infiltrating MNCs co-existed with the expression of JNK or p38 [121], suggesting that CD40 signaling or Bcl-2 family expression might be closely associated with resistance to the Fas-mediated apoptosis of MNCs in SS.

We investigated whether another molecule assists the inhibitory effect on Fas-mediated apoptosis. We observed the expression of the IAP family member XIAP (X chromosome-linked inhibitor of apoptosis protein) on acinar epithelial cells in our in vitro study of the enhancement of XIAP expression by IL-1β or TGF-β in HSG cells [122]. Because gender difference is thought to be associated to pathogenesis of SS, the presence of XIAP expression in salivary glands might influence on high frequency of SS in female gender. Although the expression of the low-molecular-weight redox protein thioredoxin (TRX) was also observed on LSGs from SS patients [123], it was reported that Fas-mediated apoptosis as well as the IFN-γ-induced expression of IL-6 in HSG cells was inhibited by recombinant TRX [124]. A relationship between epidermal growth factor (EGF) and SS was reported in both salivary and lacrimal glands [125,126,127]. EGF, which combines with EGF receptor (EGFR) to control cell development and proliferation, is secreted from salivary glands. The clinical importance of EGF in both salivary and lacrimal glands in SS has also been discussed, as a relationship between the EGF concentration and exocrine function was demonstrated [128,129,130,131].

**Figure 2:**
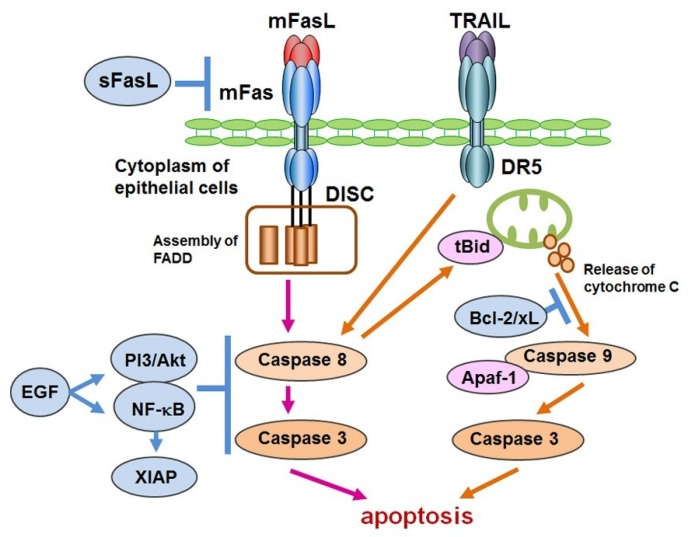
Inhibition of apoptosis mediated by mFas and TRAIL. The pathway for the induction of mFas-mediated apoptosis consists of death-inducing signaling complex (DISC) formation followed by the assembly of Fas-associated protein with death domain (FADD), and the cleavage of caspases 8 and 3. One of the inhibitory mechanisms of Fas-mediated apoptosis is mediated by epidermal growth factor (EGF) and a downstream PI3K/Akt and NF-κB pathway including the expression of X-chromosome-linked inhibitor of apoptosis (XIAP). Another candidate is sFasL, which is shown to inhibit mFas-mediated apoptosis by combining with mFas. Although pro-apoptotic pathway in SS is similar to general apoptotic pathway, EGF-mediated inhibition of apoptosis might be specific in SS. TNF-related apoptosis-inducing ligand (TRAIL) rapidly induces the apoptosis of cultured salivary gland epithelial cells (SGECs), in which a mitochondrial pathway that includes a release of cytochrome C, the cleavage of caspase 9, and the activation of Apaf-1 after the truncation of Bid is also involved. Bcl-2 family molecules such as Bcl-2 and Bcl-xL are considered to inhibit this mitochondrial pathway by inhibiting cytochrome C release.

With regard to the induction of EGF-associated molecules, we observed that EGF activated PI3K-Akt and NF-κB pathways separately with an induction of EGFR activation in SGECs by EGF as well as the in vivo expression of phosphorylated Akt in the cytoplasm and p65 in the nuclei of ductal cells of ductal cells [132]. In our study, using an experimental inhibition of Fas-mediated apoptosis in SGECs from patients with SS, an anti-apoptotic effect of EGF was identified [133]. A combination of anti-Fas antibody and LY294002 induced TUNEL-positive apoptosis in SGECs from SS patients, and EGF dose-dependently inhibited the apoptosis, showing a direct effect of EGF against Fas-mediated cell death (Figure 2).

In their investigation of EGFR signaling and Fas-mediated apoptosis, Yang et al. reported a relationship between muscarinic type 3 receptor (M3R) and EGFR in association with an apoptotic pathway mediated by the Fas/FasL system [134]. Although M3R is a one of the major autoantigens in SS [135], recombinant M3R peptide induces murine sialadenitis with M3R-reactive T-cell expansion, and a retinoic acid-related orphan receptor-gamma t (RORγt) antagonist can inhibit SS-like sialadenitis [136]. Interestingly, the immunization of M3R mimotope (which has structure similar to that of the original epitope) effectively inhibited the sialadenitis, the production of pro-inflammatory cytokines, and the autoantibody against M3R and apoptotic pathway mediated by Fas, suggesting a crucial role of EGF signaling in inflammation and apoptosis in SS.

**Figure 3:**
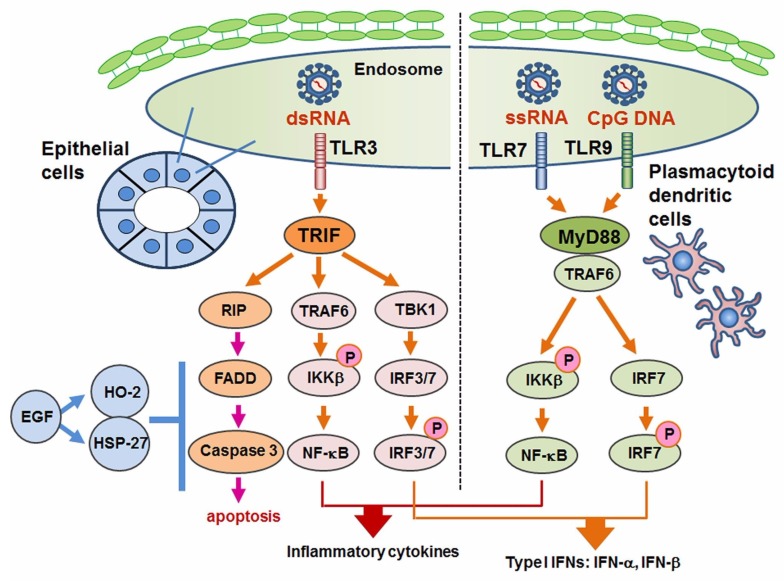
Role of TLR3, 7 and 9 in SS. Toll-like receptor3 (TLR3), which responds to double-strand RNA (dsRNA) of microbes, usually activates the NF-κB pathway and interferon regulatory factor (IRF) 3–7 downstream of Toll-IL-1 receptor domain-containing adaptor inducing IFN-β (TRIF). However, TLR3 also has the ability to induce apoptosis of SGECs by activating a RIP pathway that includes the assembly of FADD and the cleavage of caspase 3. Epidermal growth factor (EGF) and EGF-mediated heme oxygenase (HO)-2 and heat shock protein (HSP)-27 are candidates for the inhibition of TLR3-mediated apoptosis. Crucial roles are also played by TLR7 and TLR9 signaling, which respond to single-strand RNA (ssRNA) and to the CpG DNA of viruses in plasmatoid dendritic cells in exocrine glands in SS, myeloid differentiation factor (MyD) 88, and its downstream pathways including the NF-κB pathway and the phosphorylation of IRF7. NF-κB and IRF7 mediate the up-regulation of inflammatory cytokines and type I interferon (IFN) including IFN-α and IFN-β. Investigations into the detailed functions of TLR7–9 signaling in epithelial cells of patients with SS are in progress.

We investigated the expression of relevant molecules and the mechanism of cell death control in SS with respect to TLR3-mediated apoptosis [137]. Although the expression of the downstream signal of TLR3 was limited, our in vitro investigation with poly I:C showed the full activation of RIPK3, FADD and caspases in cultured SGECs from SS patients. In addition, EGF associated with the expressions of hemeoxygenease-2 and heat shock protein 27 dose-dependently inhibited TLR3-mediated apoptosis (Figure 3).

## 8. Conclusions

The induction of apoptosis in LSGs and infiltrating MNCs in SS is mediated by the Fas/FasL system as well as cytotoxic granules and innate immunity such as TLR3 signaling. Fas/FasL has both a membrane-bound form and a soluble form, these forms’ cytotoxic functions are thought to differ. Animal studies have provided useful information about membrane-bound Fas-mediated apoptosis, including findings of confounding factors such as estrogen and aging. However, the precise effect of membrane-bound Fas on apoptosis remains to be identified. Although Fas-mediated apoptosis in SS is strictly controlled by the assistance of cell-survival factors including members of the Bcl-2 family or the PI3K/Akt pathway, other factors such as an individual’s genetic background or epigenetic alteration should be investigated with respect to the relationship between cell death mediators and these background conditions. With regard to sFas/sFasL, cytotoxic granules, and TRAIL, their cytotoxic pathways including the activation of death domain and cleavage caspases have been elucidated in part with some concerns about polymorphism. Much remains to be clarified.

Recent studies also revealed the involvement of TLR signaling in SS in accordance with the progress of research into the importance of innate immunity in autoimmune diseases such as SS. The roles of TLR7–9 as sensors for nucleic acids of exogenous microbes have been revealed, whereas TLR3 has a specific role in the induction of apoptosis in SS. For the clarification of clinical conditions of SS, further research into TLR signaling for apoptosis and the induction of inflammation that is caused by the type I IFN pathway and NF-κB pathway in labial and lacrimal glands would be useful.

Taken together, the above-described findings indicate that the apoptosis of SGECs is tightly controlled by pro-apoptotic signals that center on the Fas/FasL system and anti-apoptotic signals that consist of Bcl-2 family members and/or EGF. The involvement of TLR signaling that contains an apoptotic pathway as a disease mechanism for Sjögren’s syndrome has also been discussed over the past decade. Although SGECs might be strictly protected from various pro-apoptotic signals, the research into cell-survival control is at a quite primitive stage. Biological approaches to find novel cell-survival factors in Sjögren’s syndrome are strongly required.
